# Ultrafast Fiber Bragg Grating Interrogation for Sensing in Detonation and Shock Wave Experiments

**DOI:** 10.3390/s17020248

**Published:** 2017-01-27

**Authors:** George Rodriguez, Steve M. Gilbertson

**Affiliations:** 1 Los Alamos National Laboratory, Laboratory for Ultrafast Materials and Optical Science, MS K771, Los Alamos, NM 87545, USA; 2 Los Alamos National Laboratory, DARHT Experiments and Diagnostics, MS P940, Los Alamos, NM 87545, USA; steveg@lanl.gov

**Keywords:** fiber Bragg grating, fiber sensing, shock waves, detonation, high-speed interrogation

## Abstract

Chirped fiber Bragg grating (CFBG) sensors coupled to high speed interrogation systems are described as robust diagnostic approaches to monitoring shock wave and detonation front propagation tracking events for use in high energy density shock physics applications. Taking advantage of the linear distributed spatial encoding of the spectral band in single-mode CFBGs, embedded fiber systems and associated photonic interrogation methodologies are shown as an effective approach to sensing shock and detonation-driven loading processes along the CFBG length. Two approaches, one that detects spectral changes in the integrated spectrum of the CFBG and another coherent pulse interrogation approach that fully resolves its spectral response, shows that 100-MHz–1-GHz interrogation rates are possible with spatial resolution along the CFBG in the 50 μm to sub-millimeter range depending on the combination of CFBG parameters (i.e., length, chirp rate, spectrum) and interrogator design specifics. Results from several dynamic tests are used to demonstrate the performance of these high speed systems for shock and detonation propagation tracking under strong and weak shock pressure loading: (1) linear detonation front tracking in the plastic bonded explosive (PBX) PBX-9501; (2) tracking of radial decaying shock with crossover to non-destructive CFBG response; (3) shock wave tracking along an aluminum cylinder wall under weak loading accompanied by dynamic strain effects in the CFBG sensor.

## 1. Introduction

Over the last five years, we have concentrated on developing and applying high-speed fiber Bragg grating (FBG) sensor interrogation approaches to shock wave and high explosive detonation front tracking for experimental situations where continuous position monitoring of the propagating disturbance is monitored along the length of a chirped FBG (CFBG) [1,2,3,4]. Most recently, with the use of coherent pulsed ultrafast laser-based FBG interrogation systems [5,6,7], full spectral readout of uniform non-chirped FBG sensors at 100-MHz interrogation rates has been demonstrated as a valuable diagnostic for high speed strain and pressure measurement for dynamic materials under sub-microsecond loading conditions. Important complementary work [8,9,10] using FBG sensors as shock pressure transducers for plate-impact shock wave studies have also demonstrated progress in this field. In this paper, we present a similar approach as in [5,7], but this time, we apply the approach to fully resolve (space and time) shock wave and detonation front propagation dynamics with distributed sensing using a 10 mm–100 mm-long CFBG. With the capability of serially stacking 100 mm-long C- and L-band CFBGs on a single fiber, continuous tracking of shock or detonation wave propagation out to 200 mm in length is now also possible. Full spectral response of the CFBG is stroboscopically interrogated with a broadband short pulsed laser, and a streaked record of shock position versus time along the CFBG is obtained using a coherent pulse time-stretch approach [6]. This approach differs from the previously published CFBG interrogation method described in [1,2,4] in that the detection in the time-stretch streaked approach fully resolves the CFBG spectrum and is sensitive to spectral changes in real time, whereas the original CFBG approach [11,12,13] is only sensitive to time changes in the integrated CFBG spectrum. In both approaches, the CFBG sensor still offers a common benefit to researchers seeking embedded fiber detection of dynamics when a minimally-intrusive embedded sensing method is desired or when direct line of sight optical access (i.e., streak camera, optical ranging [14], PDV [15], VISAR [16], etc.) is not possible. The clear advantage of the time streaked approach over the integrated approach is when shock pressure conditions are relatively weak (i.e., <5 GPa) as described in [5,7]. In experiments where we have demonstrated pressure conditions sufficiently low enough that the shock wave does not destructively process the CFBG, the streaked approach may offer quantifiable measurements of strain or pressure along portions of the CFBG experiencing local stress as was done for uniform FBGs for point-like measurements reported earlier [5,7].

The goal of this paper is to introduce the researcher to CFBG-based shock wave and detonation front fiber sensing and to describe the current approaches, interrogation systems and sensor characteristics available for use under this purpose. In this paper, we describe our approaches to CFBG sensing to measure detonation front and shock wave propagation dynamics [17]. In Section 2.1, we describe the two CFBG interrogation approaches we currently use: the spectrally-integrated approach and the time-streaked spectrally-resolved approach. In Section 2.2, we also detail the specifics of the CFBG sensors we have fielded in experimental tests. In Section 3, three experimental tests are described: (1) linear high explosive detonation front measurements in a rectangular slab of the plastic bonded explosive (PBX): PBX-9501; (2) shock front positional tracking of a radial decaying inert shock wave produced by a detonator; (3) shock wave tracking along a cylinder wall under weak loading accompanied by CFBG strain effects. Finally, a short discussion is given in Section 4 where we describe current spatial and temporal resolution with existing deployed systems and possible research directions for future improvements. Conclusions are in Section 5.

## 2. CFBG Recording Systems and Sensors for Detonation and Shock Wave Research

Depending on the objective of the measurement, there are several interrogation system types currently deployed and FBGs used for different purposes (i.e., shock wave tracking, detonation, pressure, temperature, etc.). Because detonation and shock fronts travel at speeds in excess of the material sound speed (we have measured shock wave phase velocity speeds above 10 mm/μs), the spatial resolution required for accurate position tracking measurements imposes an FBG interrogation speed with an equal to or better than 10-ns time resolution. As a result, much of the systems being deployed in the field are interrogators that operate in the 100 MHz to the GHz rate. We first describe our interrogation systems and then the types of FBGs used for measurements.

### 2.1. GHz High Speed FBG Interrogation Systems

#### 2.1.1. Spectrally-Integrated Approach

**Figure 1:**
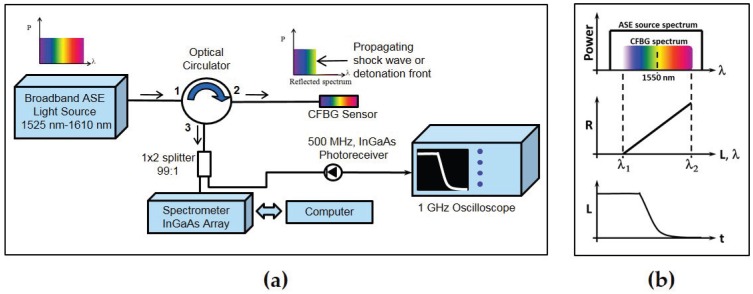
(**a**) High speed CFBG shock and detonation interrogation system based on the spectrally-integrated approach; (**b**) illustration of chirped fiber Bragg grating detonation velocity sensor system response functions. The CFBG spectrum is narrower than the ASE light source spectrum. The total reflected light intensity (R) is proportional to the grating length (L) and wavelength (*λ*). A CFBG sensor adjacent to a detonating explosive will produce data that measure the detonation position (L) as a function of time.

Our earliest systems and results [1,2] relied on the interrogation approaches developed by Benterou and Udd [11] for measuring high explosive detonation front position and velocity tracking where a linear chirped FBG (CFBG) sensor is deployed. In addition to our group, this approach has also been adopted by Barbarin et al. in France with excellent results on detonation propagation measurements on several types of high explosives [3]. This approach is illustrated in Figure 1a. An incoherent broadband (1525 nm–1610 nm) amplified spontaneous emission (ASE) fiber light source is used to continuously illuminate the CFBG sensor located on the experimental target assembly (typically, a high explosive charge). Depending on the desired detonation front run length to be measured, the CFBG sensors we have used have ranged from as short as 10 mm to as long as 200 mm. Broadband light reflected off the CFBG is then sent to a single InGaAs photodiode that integrates the total light return from the sensor. Then, as the sensor length shortens due to intense destructive shock processing from the explosive detonation front, the photodiode signal voltage decreases in proportion to the total integrated spectrum, which is linearly mapped along the length of the sensor. Thus, this approach detects temporal changes in the total integrated spectrum of the CFBG. The time resolution of the interrogation system is determined by the speed of the receiver/oscilloscope combination, typically between 1 and 2 ns. Mapping of the CFBG reflected spectrum to wavelength and length is illustrated in Figure 1b. Numerical inversion of the photodiode signal recorded waveform on the oscilloscope to CFBG length is done by post-processing the signal waveform with *a priori* knowledge of the unperturbed reflection spectrum and chirp rate of the CFBG sensor. The analysis method for determining the length/position versus time response of the grating from the experimentally-recorded trace of the voltage versus time function has been previously [1] described and is shown graphically in Figure 1b. The broadband grating reflection spectrum is centered at 1550 nm, and an ASE source is used to illuminate the entire grating bandwidth. The light intensity, R, reflected from the grating is proportional to the integrated grating spectrum return light. Because the grating is linearly chirped, R is proportional to the grating length (L) and wavelength, *λ*. The integrated light return voltage signal is,

(1) $$R\left(t\right)=a\int_{\lambda_{1}}^{\lambda_{2}\left(t\right)}S_{G}\left(\lambda \right)ASE\left(\lambda \right)d\lambda ,$$

where a is a normalization constant, S${}_{G}$(*λ*) is the grating reflection spectrum, ASE(*λ*) is the light source spectrum and $\lambda_{1}$ and $\lambda_{2}$ are the lower and upper wavelength limits, respectively. The wavelength location where the detonation front is located at time t is assumed to be $\lambda_{2}\left(t\right)$. Therefore, L(t) ∝ R(t), and $\lambda_{1}$ corresponds to the shortest wavelength reflected by the grating, which is the location nearest to the light source. Data analysis [1,11,12] yields time-of-arrival data L(t) for a detonation propagating along the CFBG sensor. The slope (velocity) is calculated by performing a numerical fit to the L(t) data to extract the local detonation phase velocity along the direction where the CFBG sensor was located. Processing of dynamic data is done using the following steps: First, the spectrum and chirp rate of the CFBG are measured before the experiment. The spectrum is numerically integrated along the *λ* axis and normalized to one. The x-axis of the integral function is then converted from *λ* to position after multiplying by the chirp rate. Therefore, a synthetic LUT (look-up table) is generated with the integral function. The dynamic data initially recorded by the oscilloscope as voltage (i.e., integrated CFBG reflection intensity) versus time is then also normalized to unity. A one-to-one mapping of dynamic normalized voltage CFBG data to position is done on a point-by-point basis in time by using the LUT to match a voltage to a corresponding position value in the LUT. The output of the post-experiment analysis yields a position versus time plot of the CFBG response under loading. The local average velocity is computed from the slope of a linear fit to this data.

#### 2.1.2. Time-Streaked Spectrally-Resolved Approach

**Figure 2:**
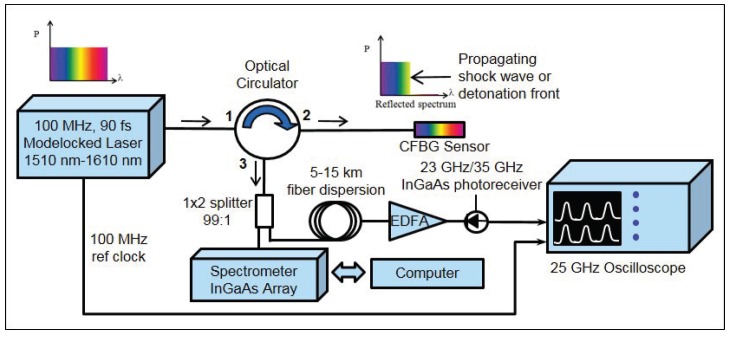
High speed CFBG shock and detonation interrogation system based on the time-streaked spectrally-resolved approach.

In some of our early experiments, we became aware of possible complications associated with the spectrally-integrated approach when shock pressures were not sufficiently high enough to destructively “process” the FBG for position information. Undesirable non-monotonic signal voltage versus time is observed when low amplitude shocks or pressure waves perturb, but do not destroy, the sensor [4,18,19]. Instead, the FBG sensor spectrum shifts due to the applied stress (or temperature) and renders the data extraction analysis described in the previous section inapplicable. Another complication with the spectrally-integrated approach is the scenario when the initial shock wave strikes and disrupts the CFBG sensor at a position other than the end tip. Without a precise measurement of the location in the CFBG spectrum at initial impact other than the end tip, accurate extraction of the position dynamics becomes difficult using the spectrally-integrated approach. To overcome these potential issues, we introduced a new ultrafast coherent pulsed approach for FBG interrogation [5,6] capable of full spectral readout every 10 ns, i.e., at 100 MHz. By spectrally resolving the dynamic response in time, determining the shock (or strain) dynamics becomes tenable. In much of our latest work, this method, which we call the “time-streaked spectrally-resolved approach”, has become our preferred approach for situations when the FBG or CFBG sensor is expected to partially survive the applied pressure loading or if impact from the shock wave is expected at a position other than the end tip of the CFBG sensor. Although we have demonstrated this approach with short 1 mm-long uniform FBGs (i.e., single-color FBGs) for point-like dynamic pressure and strain measurements [5,7], here, we describe how the approach is used to time-streak and spectrally resolve a broadband multi-color CFBG-based sensor. In Figure 2, we show a block diagram illustrating the main components of this type of interrogator. Pulses from a broadband (1510 nm–1610 nm) ultrafast 90-fs erbium mode-locked fiber laser are used to continuously strobe the CFBG at 100 MHz. Because each pulse is temporally coherent, each reflected pulse from the grating has a well-defined frequency-dependent phase velocity comprised of the spectral components in the reflected pulse. Chromatic dispersion is introduced to temporally stretch the pulse to the nanosecond regime using a long run (typically between 5 and 15 km) of Corning SMF-28e fiber to separate out spectral components using the fiber’s material group velocity dispersion properties. The average chromatic dispersion fiber constant for the fiber is 16.7 ps/nm/km at 1550 nm. After dispersion, if the return power is too weak and below the detector sensitivity, each pulse can be amplified using an erbium-doped fiber amplifier (EDFA). Pulses are detected with a high speed 23 GHz (or 35 GHz) InGaAs photoreceiver. Photoreceiver waveforms are recorded and stored with a fast 25-GHz, 100-GS/s (GS/s =10${}^{9}$ samples/sec) transient recording digitizing oscilloscope. In addition to the CFBG streaking signal, a 100-MHz laser clock pulse is also recorded by the oscilloscope as a timing reference clock used to aid post-shot analysis by serving as a stable time-tie mark for every pulse recorded during a shot. Because the full spectral response of the CFBG is strobed with the pulsed laser, a streaked record of shock position versus time along the CFBG is obtained. This approach differs from the interrogation method in the previous section above. *The detection in the streaked approach fully resolves the CFBG spectrum and is sensitive to changes in real time, whereas the spectrally-integrated approach is only sensitive to time changes in the integrated CFBG spectrum*. The time resolution of the time-streaked interrogation system is determined by the inverse time of laser repetition rate, 10 ns.

**Figure 3:**
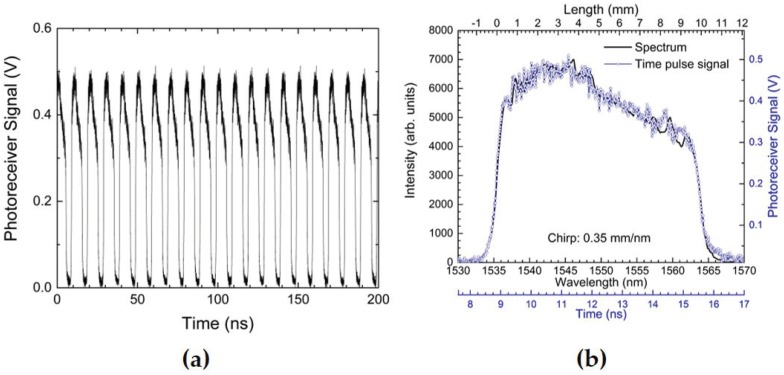
(**a**) A short 200-ns sample of a time-streak CFBG waveform showing the 100-MHz pulse train detected by the 35-GHz photoreceiver and recorded by the oscilloscope after the laser has interrogated a 10 mm-long C-band CFBG; (**b**) a single pulse (blue trace) from the waveform in (**a**) shows that the pulse has been chromatically dispersed and time stretched to 6.7 ns using a 13 km-long spool of fiber; the time axis sample rate is 100 GS/s. A measure of the time integrated CFBG spectrum (black trace) from the arrayed spectrometer matches the shape of the time-streaked pulse, and the CFBG length is indicated in the top axis of (**b**).

In Figure 3, we show example waveforms that aid in understanding time domain waveform signals from a time-streaking interrogation system. The example given is for a 1535 nm–1565 nm, linearly-chirped, C-band 10 mm-long CFBG grating centered at 1550 nm. The CFBG chirp rate is 0.35 mm/nm. Figure 3a shows a very short 200-ns segment of time-streaked CFBG data as recorded by the oscilloscope. The interrogation rate is 100 MHz, as determined by the repetition rate of the mode-locked laser. In Figure 3b, we zoom in to a single pulse (blue trace) showing a time stretch to 6.7 ns after chromatic dispersion has been applied using a 13-km spool of SMF28e fiber. The sample rate of the time axis is 100 GS/s as determined by the oscilloscope sampling speed. High bandwidth electronics are necessary to adequately time resolve the spectral-temporal properties of every single pulse. The slowest component in the detection system is the digitizing oscilloscope at a 25-GHz bandwidth (i.e., 40 ps). The inter-pulse time is 10 ns. Therefore, the effective number of points resolved in a 10-ns window is n${}_{pts}$ = 250 points (n${}_{pts}$ = 10 ns/40 ps). We can also approximate the effective spectral resolution, R${}_{\lambda }$, of the time-streak waveform in Figure 3b by knowing the fiber dispersion at D${}_{f}$ = 16.7 ps/nm/km and the total fiber dispersion length used (L${}_{D}$ = 13 km) in a Δt${}_{w}$ = 10 ns time window as,

(2) $$R_{\lambda }=\left(\Delta t_{w}/n_{pts}\right){\left[L_{D}D_{f}\right]}^{-1}.$$

Plugging in the values above yields R${}_{\lambda }$ = 0.18 nm as the effective spectral resolution in the single pulse time-streak data of Figure 3b, and the sampling resolution at 10 ps per point is 0.045 nm. The resolution can be varied by changing the total length of fiber dispersion. Post-experimental analysis to convert the time domain streaked waveform of Figure 3b to CFBG length is done by first converting the time axis to wavelength followed by scaling the spectrum to CFBG length using the known chirp value for the grating. For comparison to the time-streaked waveform plot, in Figure 3b, we also overlay a plot of the time-integrated spectrum (black trace) of the same CFBG using the arrayed spectrometer. The top x-axis of Figure 3b also shows the CFBG length after multiplying the wavelength axis by the CFBG chirp, 0.35 mm/nm. Very good reproducibility between the time-streaked and integrated spectra is shown, giving us confidence in the time-streaked approach to interrogating FBGs.

### 2.2. Characteristics of Fiber Bragg Gratings for Detonation and Shock Physics Measurements

In our applications, we have used a variety of single-mode Type-I CFBG sensors with the shortest CFBG being 10 mm and the longest at 200 mm. All of the CFBGs are phase mask UV written on Ge-doped Corning SMF28e step-index-type fiber. The nominal fiber index is *n* = 1.4682. In addition, we used polyimide and acrylate-coated versions with a recoat process after the grating is written. For detonation fronts and strong shocks, the effects of coatings over the CFBG portion can be neglected due to the high pressures (and temperatures) present during destructive shock processing of the CFBG. Even when shock pressures are sufficiently low that an elastic strain response is present, the strains are still very high, exceeding 10${}^{4}$ μϵ, and it may be possible that the coating may be contributing. However, shock studies of FBG elastic response under conditions of high strain and high strain rate as in a shock wave are the subject of on going work [8,9,10]. Table 1 lists the various CFBGs used in this work. Typically, the C-band gratings are centered at *λ* = 1550 nm and are greater than 95% reflective across the wavelength range *λ* = 1533 nm–1568 nm. The C-band CFBGs are designed to fit within the flattest portion wavelength range of our ASE and mode-locked fiber laser sources. In addition to C-band CFBG, broader spectral C + L-band CFBGs are also used to increase spatial resolution or extend the CFBG length, for example to 200 mm-long sensors. The C + L-band CFBGs cover a wavelength range *λ* = 1533 nm–1595 nm and, therefore, also require broadband light sources that extend to the long wavelength end of the L-band, *λ* = 1605 nm. The vendors used for our custom-made CFBG sensors are Timbercon, Inc. (Tualatin, OR, USA), and O/E Land, Inc. (La Salle, QC, Canada).

**Figure 4:**
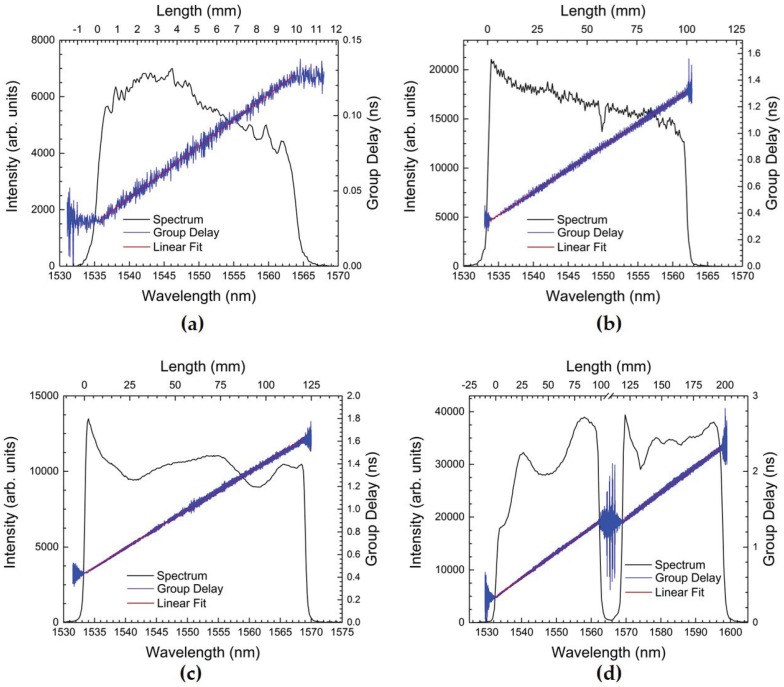
Spectrum (black), group delay (blue) and linear fit (red) plots for various CFBG sensor lengths: (**a**) 10 mm, (**b**) 100 mm, (**c**) 120 mm and (**d**) 200 mm. The 200-mm CFBG in (**d**) consists of two 100 mm-long gratings (C-band and L-band) physically separated by 1 mm. The slope of the linear fit to the OBR measured group delay data plots yields the chirp for each grating. The fitted slopes are: (**a**) 0.00344 ns/nm, (**b**) 0.0342 ns/nm, (**c**) 0.0334 ns/nm and (**d**) 0.03347 ns/mm (C-band) and 0.03443 ns/nm (L-band). After conversion to units of mm/nm, the linear chirp values are: (**a**) 0.351 mm/nm, (**b**) 3.49 mm/nm, (**c**) 3.41 mm/nm and (**d**) 3.42 mm/nm (C-band) and 3.51 mm/nm (L-band).

Table 1 also lists the chirp rates used for the CFBGs. Depending on the chirp rate, we previously demonstrated that the positional sensitivity of the sensor can be adjusted [4]. The chirp of each grating used is measured before installation on an experimental test. The chirp is measured using a Luna Innovations frequency-swept optical backscatter reflectometer (Model No. OBR 3600). This ensures that every CFBG sensor chirp rate and length is calibrated before use. In Figure 4, we plot the reflection spectra and OBR measured chirp for several CFBGs. The Luna instrument measurement of the chirp on the same CFBG is repeatable to within 0.1%. However, due to manufacturing variability, LUNA OBR measurements of the chirp on CFBGs of the same fiber type can vary as much 1.5% from the design specification. We therefore characterize and measure the chirp of each CFBG using the Luna OBR and then use measured chirp for each individual CFBG to analyze its corresponding dataset. Often, we must trim the CFBG to length to match the experimental assembly, and we accomplish this using a femtosecond laser machining apparatus [1]. The OBR utilizes a frequency-swept laser interferometer to scan across the entire band of the CFBG and outputs 2× the group delay, because it measures the wavelength-dependent group delay in double pass. The slope of a linear fit line to the OBR group delay measurement is used to extract the chirp in units of ns/nm. To convert from units ns/nm to linear chirp in length per unit wavelength (i.e., mm/nm), we multiply the group delay slope by the factor (0.5 c/n), where c = 299.792 mm/ns is the speed of light and *n* = 1.4682 is the average fiber index. The 0.5 factor is to account for the fact that the Luna OBR measures the group delay in double pass.

After chirp calibration of the sensors, they are appropriately then trimmed in length to fit according to the experimental assembly requirements. Direct bonding of the CFBG sensor is done with a cyanoacrylate adhesive (M-Bond 200 from Vishay Precision Group, Inc.) that is compatible with most materials, including many of the plastic bonded explosives we have used in our tests. After bonding the CFBG sensors, we accurately measure the CFBG tip position to register a location on the device so that post-analysis hydrodynamic flow calculation models have a dataset for comparison. Typically, we measure the start tip position of each sensor to ±25 μm.

## 3. Experimental Tests

Three experimental test results using our high-speed CFBG sensing systems are described. In the first example, we demonstrate full detonation front position versus time measurement using both the spectrally-integrated approach and the time-streaked spectrally-resolved approach. The CFBG measurement was compared against several other complementary discreet time-of-arrival diagnostics. In the second example, we present data showing the effect of a radial shock decay initiated by a detonator. As the shock wave decays to a high-pressure wave, crossover from destructive mode shock processing to a non-destructive high-pressure wave interacting with the CFBG is studied. It shows a case when only the time-streak spectrally-resolved approach would be adequate to determine the position of the disturbance. As a final example, we then present an experimental situation where the shock pressure driving the CFBG is weak, such that a complex elastic strain response coupled to the shock wave propagation is observed. The CFBG sensors in this case are used to monitor shock transit along an aluminum hollow cylinder wall and is a test case where only the time-streak spectrally-resolved approach is capable of separating out shock position from elastic strain response effects.

### 3.1. Linear Detonation Front Measurements in the Plastic Bonded Explosive PBX-9501

An 8 inch-long × 8 inch-wide × 0.5 inch-thick slab of the plastic bonded high explosive (HE) PBX-9501 was uniformly initiated along one edge using an explosive line wave generator (LWG) [20]. The line wave generator is started at its apex point with a single SE-1 detonator. The goal of this test was to cross compare the performance of a series of detonation front position diagnostics along the top surface of the main charge. The nominal detonation velocity for PBX-9501 is 8.8 mm/μs. The time-of-arrival electrical pin, electrical flat cable shorting switches, piezo pin and CFBG diagnostics were used to record the detonation front as it transits the length of the main HE charge. The time of arrival electrical shorting pins and piezo pins have been the standard for shock and detonation discreet position transducer measurements for many decades. They operate as coaxial electrical probes that incorporate a switch at the tip that produces an electrical signal when impacted by the detonation or shock front. The switch at the end of the probe may consist of a non-biased piezo crystal (piezo pin) or a voltage-biased unterminated air gap (shorting pin) that is crushed by the intense shock pressure to produce a high speed voltage transient indicative of the arrival of the shock front. The signal voltage is self-generated in the piezo pin under shock compression, and the bias voltage of the shorting pin was set to 200 V. The response time of these pins is 10 ns. The piezo and electrical shorting pins were procured from Dyansen, Inc. (Goleta, CA, USA), and the part numbers were CA-1037-C and CA-1135-SL, respectively. The electrical flat cable shorting switches are custom-made probes that operate in a similar fashion as the shorting pins, but instead have their transmission line and an array of metal disc pads embedded in a flat cable comprised of a multilayer cable strip of Kapton polyimide and copper. The overall thickness of the flat electrical shorting switches is only 0.003”, and the active metallic square shorting pads where the sensing occurs are only 200 μm${}^{2}$ in area with a 110-μm gap between the switch and grounding plane. The bias voltage for the shorting switches was 180 V, and the response time of each switch in the linear array is ∼4 ns.

**Figure 5:**
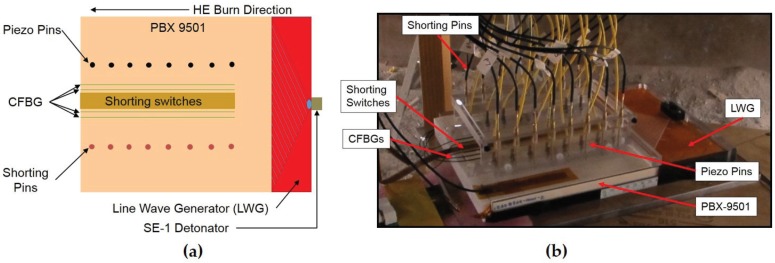
(**a**) PBX-9501 slab test illustration showing the location of the various detonation front time-of-arrival diagnostics; (**b**) a photograph of the assembled test indicating the locations of the various diagnostics.

In Figure 5a, we illustrate the geometry of the slab detonation experiment, and in Figure 5b, we show a photograph of the assembled experiment showing various locations of the diagnostics. A total of four 120 mm-long CFBGs (nominal chirp of 3.4 mm/nm) were fielded with two CFBGs straddling the center line on either side of a 16-point linear strip array of shorting switches. Two of the CFBGs were recorded using the spectrally-integrated approach, and two were recorded using the time-streak method. The CFBGs are laid into a 0.010” machined groove in the HE, and each CFBG is fixed in place using M-Bond 200 quick setting epoxy that is chemically compatible with PBX-9501. Careful measurements of the registration of the CFBG starting tip positions relative to the PBX-9501 and LWG edge were done using a microscope-equipped height gauge with a high resolution CCD imaging camera. Similar metrology of the other single-point time-of-arrival diagnostics was also done. Table 2 lists the various diagnostics fielded for inter-comparison. All pin and switch type diagnostics are point measurements, so an array of point-type measurements is used to measure detonation front position versus time. Only the CFBG yields a continuous measure of position versus time. Figure 5a shows the relative locations of the point-type measurements, and Table 2 shows the number of point measurements for each.

**Figure 6:**
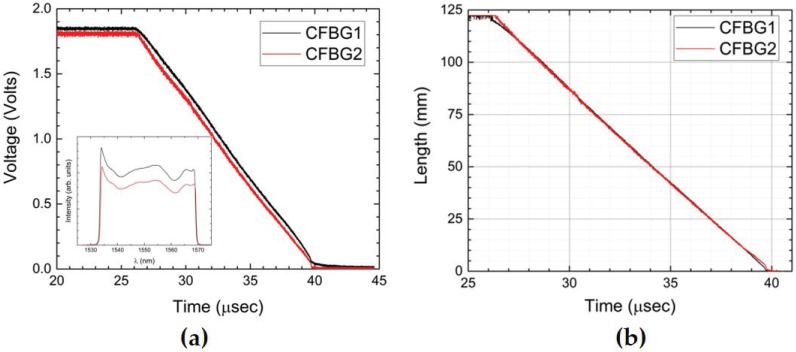
(**a**) PBX-9501 slab test showing the two CFBG signal voltage traces versus time results. The inset is a graph of showing the CFBG pre-shot reflection spectra that is needed for the data analysis; (**b**) Extraction analysis using a LUT approach allows for mapping of the signal voltage versus time to plots of CFBG length versus time.

Figure 6a is a plot of the raw CFBG voltage versus time data recorded for the experiment for the two channels using the spectrally-integrated approach. The inset to Figure 6a is the CFBG spectra associated with each 120-mm CFBG channel. The nearly linear monotonic decrease in the detector voltage versus time is indicative of a detonation front that is traveling at a constant speed during the destructive process of the CFBG. Small deviations from linearity are a result of the non-flatness of the CFBG spectrum (see the inset to Figure 6a) that must be taken into account in the analysis. The plots in Figure 6b are the extracted CFBG length versus time during the transit of the detonation along the CFBG after appropriate data processing using the dynamic voltage trace, pre-shot measured spectrum and chirp value for each CFBG. We use the look-up table (LUT) data analysis method described previously [1] for the spectrally-integrated approach to map the measured voltage to the CFBG length. In the main portion of the detonation burn, CFBG1 and CFBG2 are in excellent agreement, but small deviations at the beginning and end of the runs indicate possible mechanical decoupling of the CFBG tip from the HE at the beginning the detonation and at the end where the CFBG sensor transitions to normal bare fiber inside of loose furcation tubing.

**Figure 7:**
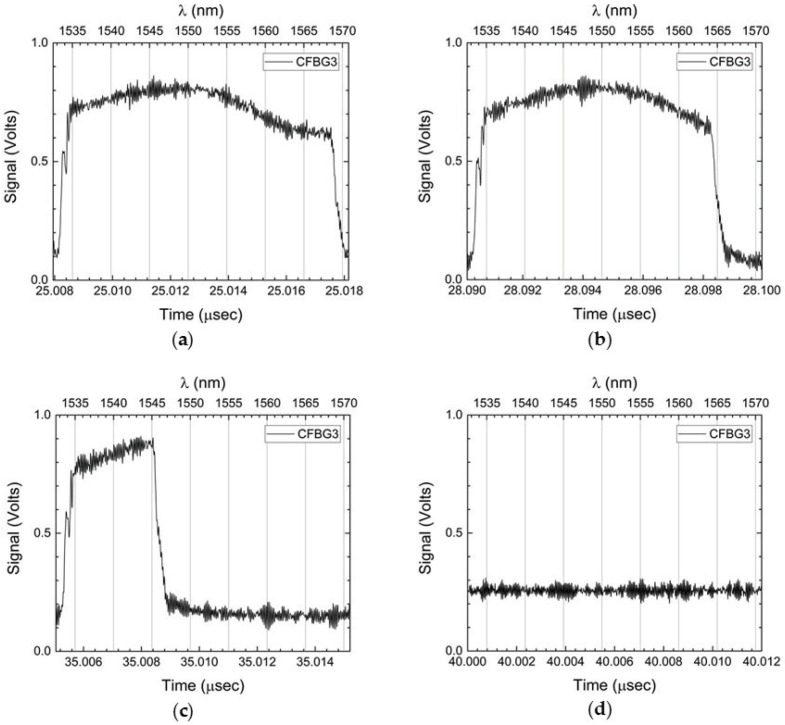
PBX-9501 slab test showing one (CFBG3) of the 120 mm-long CFBG time-streak signal traces recorded at various 10-ns time slice windows: (**a**) *t* = 25.00 μs, prior to the arrival of the detonation front; (**b**) *t* = 28.09 μs, the detonation front reaches the beginning of the CFBG; (**c**) *t* = 35.01 μs, the detonation front approaches the end of the CFBG; (**d**) *t* = 40.00 μs, the detonation front is past the end of the CFBG. Note that in each plot, the top axis has been converted to wavelength.

**Figure 8:**
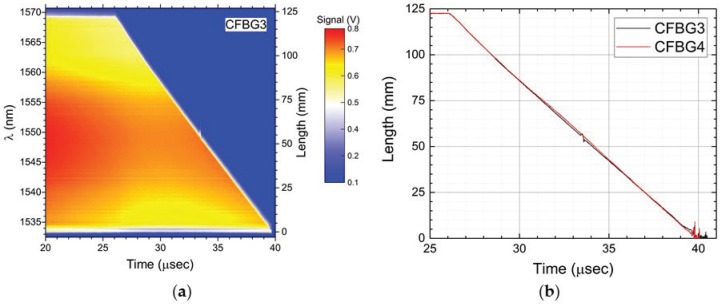
(**a**) 2D time-streak contour plot of PBX-9501 detonation propagation along the length of a 120 mm-long CFBG (3.43 mm/nm chirp). The spectral wavelength band of the CFBG is indicated on the left y-axis and the length on the right y-axis. The intensity z-axis is the signal level of the recorded output voltage of the InGaAs photoreceiver. (**b**) A pulse-to-pulse edge extraction analysis from the data in (**a**) is used to determine the position of the detonation front. The plot shows both of the 120 mm-long CFBGs (CFBG3 and CFBG4) fielded on the PBX-9501 slab test.

**Figure 9:**
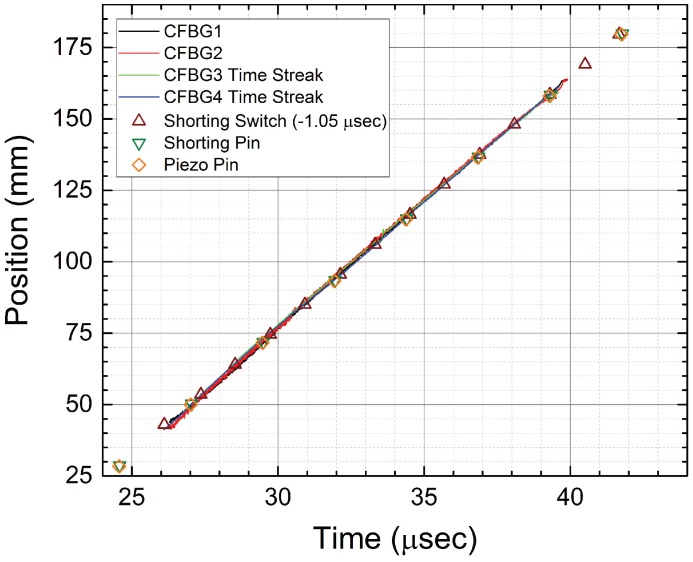
PBX-9501 slab test results plotted for all of the HE detonation diagnostics fielded. The detonation velocity and uncertainty measured for each diagnostic are listed in Table 3.

After showing the results for the two CFBGs that were fielded using the spectrally-integrated approach, we now show the results for the two 120 mm-long CFBGs that were fielded using the time-streak spectrally-resolved method. In Figure 7, we show four different 10-ns time slice windows for a single CFBG as recorded by the 25-GHz high speed digitizer (see Figure 2): Figure 7a, prior to arrival of the detonation front; Figure 7b, the detonation front reaches the beginning of the CFBG; Figure 7c, the detonation near the end of the CFBG; and Figure 7d, the detonation front past the end of the CFBG where it is fully destroyed. The time slice windows are 10 ns wide due to the 100-MHz interrogation rate determined by the mode-locked laser repetition rate. A fiber dispersion spool of 13 km of Corning SMF28e fiber was used to disperse the pulse reflected off the CFBG to a time-stretched spectrum fully contained in the 10-ns time slice window. For all of the plots in Figure 7, the top axis shows the wavelength band of the CFBG for 13 km of dispersion using the SMF28 fiber dispersion constant of D${}_{f}$ = 16.7 ps/nm/km. Post experiment data processing is used to convert every FBG interrogation time-slice window for the entire digitizer record into a 2D color contour plot of spectrum versus time. We use the recorded 100-MHz laser clock pulse as a reference inter-pulse timing fiducial to subdivide the entire CFBG waveform data record (which contains one thousand 10 ns-wide time-slice interrogation windows per 10 μs recording period) into a series of time-resolved CFBG spectra, each separated by 10 ns. A sample contour plot of this 2D time-streak spectrum for the PBX-9501 slab detonation test (CFBG3) is shown in Figure 8a. The left y-axis of Figure 8a is the spectrum of the CFBG extracted for every time slice window (as was shown on each of the top x-axes of Figure 7). The right y-axis is the CFBG length that is calculated by multiplying the CFBG spectral bandwidth by the chirp rate value (3.43 mm/nm from Table 2). The intensity, or z-axis, is the recorded signal voltage level of the photoreceiver. The x-axis is time with a resolution of 10 ns determined by the 100-MHz interrogation rate of the laser. The y-axis, however, has been sampled at 100 GS/s, and at a 25-GHz signal recording bandwidth, the spectral resolution is estimated to be R${}_{\lambda }$ = 0.18 nm (using Equation (2)). An estimated spatial resolution of 0.62 mm is obtained after multiplying R${}_{\lambda }$ by the CFBG chirp. The PBX-9501 detonation front propagation along the length of the CFBG is clearly seen as a time-dependent reduction in the bandwidth of the CFBG beginning at *t* = 26.1 μs and reaching the end of the grating *t* = 39.7 μs. The detonation front position versus time is extracted by processing the 2D contour plot using a pulse width detection method at every interrogation time sample of the x-axis. Figure 8b contains plots of the length versus time results for both time-streak CFBGs that were fielded on the PBX-9501 slab test (CFBG3 and CFBG4). Comparison of the two time- streak CFBGs shows a slight excursion from overlap between *t* = 31 μs and *t* = 35 μs that we attribute to possible error in the edge extraction algorithm when processing the 2D time-streak spectra contour plots. Placing the entire dataset (CFBG and point-type) results on a single plot allows for inter-comparison of all diagnostics listed in Table 2. All of the data plotted in Figure 9 have been corrected for placement position on the PBX-9501 and their respective associated internal timing delays relative to SE-1 detonator initiation of the LWG. All data show excellent agreement of position versus time for detonation front position in the main HE charge, except for a peculiar timing anomaly in the shorting switch data that appears 1.05 μs too late compared to the other diagnostic datasets. The shorting switch data have been globally shifted by −1.05 μs to come into agreement with the rest of the datasets. The ordinate axis is the position on the main PBX-9501 charge where zero position is at the LWG/PBX-9501 interface. The entire length of the PBX-9501 slab is 8 inches (203.2 mm), and the first point sensors are located beyond 1” (25.4 mm) to allow for any overdrive of the PBX-9501 to dissipate before a detonation position is recorded. The CFBG sensors were limited to 120 mm in length, and therefore, Figure 9 shows that they only cover a portion of the entire detonation run position, i.e., from approximately 40 mm to just over 160 mm. Although pre-shot metrology of the measured uncertainty in the placement position of the electrical and CFBG diagnostics is ±25 μm, the dynamic uncertainty of the position in the discreet electrical diagnostics is dominated by the time response when encountering a detonation front as it traverses the probe. For example, if the shorting or piezo pin response time is 10 ns, then for the detonation front traveling at 8.8 mm/μs, the position uncertainty can be as large as 88 μm. Similarly, for the shorting switches with a response time of a ∼4 ns, the uncertainty is 35 μm. The CFBG position uncertainty is determined by the scatter level in the data of Figure 9 and can be estimated as ±200 μm. The accepted value for a PBX-9501 detonation velocity in a rectangular slab is 8.80 mm/μs [20]. Table 3 is a compilation of the extracted detonation velocity taking the entire dataset for each diagnostic. The listed CFBG average velocity and uncertainty in the table is calculated from four independent slope measurements for each CFBG. The CFBG data appear to be approximately 0.45% above the accepted value.

### 3.2. Tracking of a Radial Decaying Shock: Crossover to Non-Destructive CFBG response

**Figure 10:**
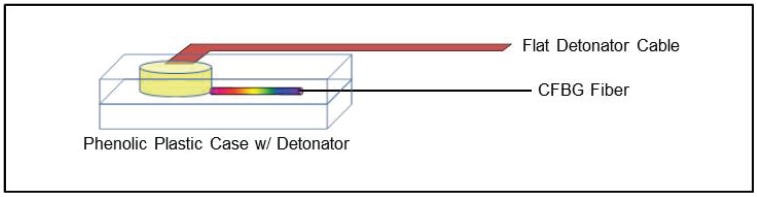
Figure illustrating the geometry used to generate a radial shock wave in a phenolic plastic case driven by the impulse from a LoFi (low fidelity) detonator.

In a recent paper [4] we presented CFBG-based HE detonation front position measurements for a variety of high explosives consisting of a cylindrical stack of various HE-types in the form of a multi-HE rate stick with a final stage of shock wave tracking into an inert material section of PMMA (polymethyl methacrylate) plastic. The measurement of the strong shock that was launched by the final HE stage into the PMMA rapidly attenuated due to the unsupported drive from the HE after reaching the PMMA. Measurements using the spectrally-integrated CFBG interrogation approach were inadequate at tracking the full shock position versus time because for low shock pressures (i.e., *p* ≤ 5 GPa), the CFBG is not destructively processed and renders CFBG shock tracking measurement ineffective. Whereas measurements of detonation-based destructive-mode CFBG tracking of position are robust with the spectrally-integrated and time-streak spectrally-resolved interrogation approaches, accurate tracking is much more subtle and dependent on peak pressures when tracking inert shocks. A more detailed approach to measure CFBG response to weak shocks that perturb, but do not destroy, the CFBG are needed to correlate sensor behavior with shock dynamics. In these cases, we assert that the time-streak spectrally-resolved approach is essential, because this interrogation approach measures the full spectral response of a CFBG under a purely strain-induced effect. Here, we demonstrate an example situation where a decaying shock wave is generated with an impulse provided by the detonator that is then allowed to radially expand in a plastic case, while losing energy from the initial impulse. The 10 mm-long CFBG records the radial shock propagation, but then at a late time exhibits a strain-induced spectral blue shift once the pressure is sufficiently low enough and is no longer able to destructively shock process the CFBG. An illustration of the geometry used for this test is shown in Figure 10. A short (10 mm long) C-band CFBG is sandwiched between two plates of phenolic plastic. A 0.010” groove is machined into the bottom plastic case to accommodate the CFBG and bare single-mode fiber. The CFBG tip is placed at the outer radius of the LoFi (low fidelity) detonator (7.7 mm diameter), and the detonator is recessed into a hole of the upper plastic plate. The CFBG spectrum measured by the time-streak method and spectrometer is shown in Figure 3b. The time-streak spectrum is generated using 13 km of SMF28e fiber for chromatic dispersion, and the CFBG chirp is 0.35 mm/nm.

**Figure 11:**
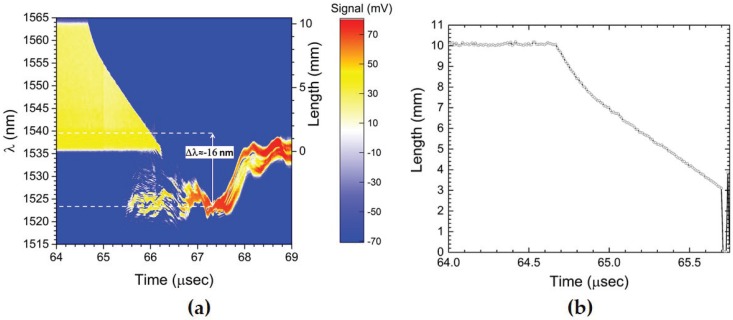
(**a**) 2D time-streak contour plot of a radial decaying shock wave and eventual crossover to non-destructive CFBG response after shock loading from a detonator-driven impulse. The spectral wavelength band of the CFBG is indicated on the left y-axis and the length on the right y-axis. The intensity z-axis is the signal level of the recorded output voltage of the InGaAs photoreceiver. A short segment of the CFBG is observed to survive at late times. At *t* = 65.6 μs, the switch over to elastic response is observed with the weakened pressure wave that introduces compressive strain in the CFBG, amounting to a peak transient induced strain of 0.013 *ϵ*. (**b**) Plot of the CFBG length versus time plot after processing the data from (**a**). The trace represents the shock location in the grating. The average uncertainty in position is ±0.075 mm.

In Figure 11a, we plot the 2D time-streak color contour plot over a 5 μs-long time window that displays the ensuing dynamics. On the left y-axis is the wavelength band of the CFBG detected and on the right y-axis is the corresponding length (or position) of the CFBG after applying the chirp value to the wavelength band. The destructive shock processing of the 10 mm-long CFBG is clearly visible, starting at *t* = 64.65 μs, where the shock strikes the CFBG tip and proceeds in a curved nonlinear fashion to near complete consumption of the grating near *t*≈ 66 μs. However, it is clearly visible that after the shock transits the entire length, a small portion (∼2–3 mm) of CFBG remains intact for times *t* > 66 μs. The data support the explanation that as the shock pressure wave decays while moving away from the detonator/CFBG tip location, it weakens to the point where that it is no longer able to destructively process the CFBG. Instead, a portion of the CFBG survives and undergoes a blue shift as the weak shock pressure briefly compresses the final few mm portion of the grating. Although not shown, for very late times, there appears a series of complex relaxation oscillations over tens of microseconds that extend beyond the recording time window that shows a small portion of the CFBG in the 1537.5 nm–1539.2 nm range remaining reflective at the end of recording. The nonlinear shape of the shock position versus time is characteristic of a slowing wave as it attenuates. In Figure 11b, we show a line-symbol plot of the CFBG length versus time plot after processing the data from Figure 11a. The trace represents the shock location in the grating, and there is a breakdown in the recovery of the shock position near L = 3 mm when interference from the CFBG’s elastic wavelength shift response prevents clean extraction of the shock position. Each symbol is separated by 10 ns, the time resolution of the time-streaked interrogation system determined by the inverse time of laser repetition rate. The scatter of the data points in the ordinate axis during the pre-shock arrival time (i.e., *t* < 64.65 μs) yields a measure of the position uncertainty after analysis. The average uncertainty in position is ±0.075 mm. The initial slope at shock entry (at *t* = 64.65 μs) is nearly 12 mm/μs. The first evidence of the spectral blue shift occurs at the position of 3.6 mm (at *t* = 65.6 μs), and the wave speed has slowed to near the sound speed of silica fiber: c${}_{o}$ = 5.6 mm/μs and in the expected range of plausible slope (velocity) measurement error of less than 2%. Therefore, we conclude that as the initial shock wave attenuates to acoustic conditions, the fiber survives and is no longer destructively processed, and instead, its elastic properties result in the dominant response at late times. Elastic strain sensing in silica single-mode uniform FBGs is known to have a strain-induced (*ϵ*) Bragg wavelength shift of −1.2 pm/μϵ and a pressure-dependent sensitivity of −4 pm/MPa under compression [5]. The mean global wavelength blue shift in the data of Δλ = −16 nm at *t* = 67.5 μs would indicate that the CFBG is sensing a peak strain and pressure of 0.013 *ϵ* and 4 GPa, respectively. The results here demonstrate that a high-speed strain/pressure measurement with CFBG sensors is possible if the pressures are sufficiently low enough to avoid destructive mode shock processing.

### 3.3. Shock Wave Tracking along a Cylinder Wall under Weak Loading Accompanied by CFBG Strain Response Effects

**Figure 12:**
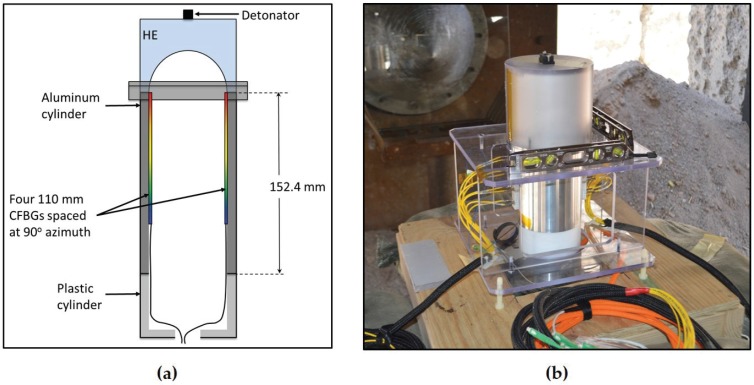
(**a**) Illustration showing the relative placement of the CFBG sensors on the inside wall of the aluminum cylinder; (**b**) a photograph of the assembled target on the firing point.

**Figure 13:**
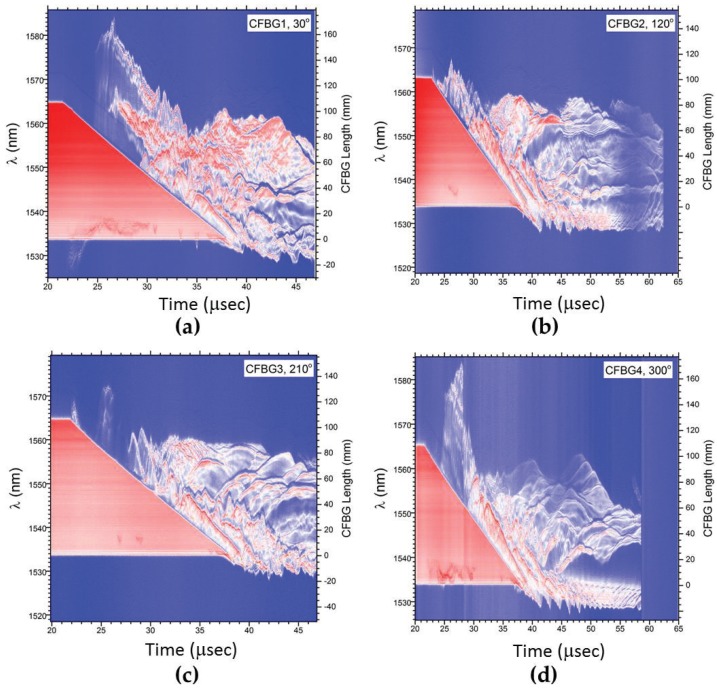
2D time-streak contour plots of the four CFBGs fielded on the cylinder wall shock wave tracking test. The CFBG and corresponding azimuth locations are: (**a**) CFBG1, 30${}^{\circ }$, (**b**) CFBG2, 120${}^{\circ }$, (**c**) CFBG3, 210${}^{\circ }$ and (**d**) CFBG4, 300${}^{\circ }$. Because of the recording system used, we note that CFBG1 and CFBG3 only record to *t* = 47 μs, and CFBG2 and CFBG4 record over a longer time window to *t* = 65 μs.

In our final example, we show CFBG results from an experimental test with a set of four ∼100 mm-long CFBGs mounted axially on the inside surface wall of a 6.1”-long (154.9 mm) by 4.75” (120.6 mm) outer diameter aluminum hollow cylinder (11.6-mm wall thickness). An axial propagating shock along the length of the wall is generated after loading from an HE PBX-9501 charge. Figure 12 illustrates the geometry used for this test. The HE drives a shock impulse into the wall of the hollow cylinder from one end. The four CFBGs were mounted such that their end tips were placed within 0.010” of the cylinder end closest to the HE (except for CFBG2, which was 0.31” away from the HE/Al interface). The four CFBGs are placed at azimuth angles separated by 90${}^{\circ }$ (i.e., CFBG1 at 30${}^{\circ }$, CFBG2 at 120${}^{\circ }$, CFBG3 at 210${}^{\circ }$ and CFBG4 at 300${}^{\circ }$). A photograph of the target assembly on the firing point is shown in Figure 12b. Anticipating a decaying shock structure because of lateral (radial) release and rarefactions inside the aluminum wall, we therefore fielded all four CFBGs using the time-streak spectrally-resolved approach. Figure 13 shows the 2D time-streak contour plot results for all four CFBGs (CFBG1–4). All of the CFBG results show a complicated response structure that would not have been otherwise possible if one had recorded the CFBG response using the spectrally-integrated approach. The CFBG data in Figure 13a, CFBG1, Figure 13c, CFBG3, and Figure 13d CFBG4, show that the detonation exits the HE, and the ensuing shock enters the aluminum cylinder at *t* = 21.5 μs. Since CFBG2 (Figure 13b) is pulled back by 0.31” from the HE/Al interface, the shock arrives at this sensor slightly delayed at *t* = 22.8 μs. In all of the data of Figure 13, although there is spectral-temporal signature of dynamic spectral shifting along much of the CFBG sensor-caused strain effects, the leading edge of the shock wave is clearly resolved if we properly analyze the 2D time-streak information. The shock-induced index modification of the CFBG allows for the detection of the edge of the shock position, but there is also large red shifting in large portions of the CFBG spectral band that can only be attributed to tensile strain in the sensor that we hypothesize is a result of the post shock release mechanics of the aluminum cylinder (i.e., initial stages of buckling) caused by lateral waves generated in the cylinder walls. Another possibility for the tensile strain observed behavior is the surface delamination process of the sensor as the M-Bond 200 adhesive used to cement the CFBGs to the cylinder yields to release the CFBG after the leading edge of shock has transited a localized shock processed region. To better understand the tensile strain dynamics, it would be useful to perform follow-on experiments with CFBG sensors located on both the inside and outside surfaces of the cylinder wall so that the wave exiting the inside and outside surfaces is simultaneously tracked. If we focus on the largest red shift that appears on channels CFBG1 (Figure 13a) and CFBG4 (Figure 13d) at *t*∼ 28 μs, an estimate of the largest strain measured by the CFBG is extracted. The largest CFBG1 and CFBG4 measured spectral red shifts are Δλ = 27 nm and Δλ = 30 nm and occur at *t* = 26.4 μs and *t* = 27.9 μs, respectively. Using the known elastic strain-wavelength shift response coefficient of 1.2 pm/μϵ yields a peak strain in the fiber of 0.023 *ϵ* and 0.025 *ϵ* for CFBG1 and CFBG4, respectively. After achieving peak strain, significant portions of the post shock regions in all of the CFBGs remain reflective (i.e., not destroyed). When the shock front reaches the end of each of the CFBGs at *t*∼ 37.5 μs, the shock pressure places each grating in a compressed state with an average spectral blue shift of Δλ∼−5 nm. Using the known pressure to wavelength shift conversion coefficient of −4 pm/MPa, an average peak pressure of 1.25 GPa is measured at the end of run in each grating. Late time signals are recorded to near 60 μs when it appears that all of the signal is lost due to fiber breakage.

**Figure 14:**
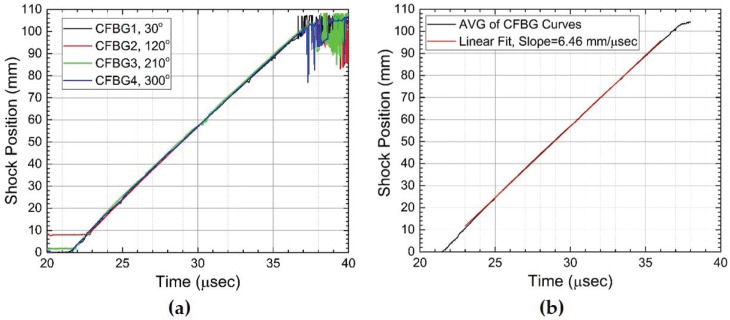
(**a**) Plots of the CFBG extracted (data from Figure 13) shock position versus time for all four of the CFBGs that were fielded on the aluminum cylinder wall test. Point-to-point position uncertainty is estimated at ±150 μm. (**b**) A shock position plot versus time shows the average of all four CFBG curves from (**a**). A least squares linear fit is also shown in red, and the slope of 6.46 ± 0.024 mm/μs is the measured average shock speed along the aluminum cylinder wall.

As was demonstrated for linear detonation front data of Figure 8 in Section 3.1, we also process the 2D contour plot data of Figure 13 to extract the shock wave position versus time. Despite having a complex spectral response, the time-streak spectrally-resolved approach is capable of identifying a precise measure of the shock position. This would not be true of the spectrally-integrated approach where undestroyed portions of the grating would lead to large uncertainty in the position measurements. In Figure 14a, we overlay the results of the CFBG shock position versus time line plots for the four CFBGs fielded. Excellent agreement in the dataset is observed. Late time CFBG shock position data after *t* = 37.5 μs are difficult to resolve because of complications from the pressure-induced spectral shift that makes pulsed edge detection impossible. Nonetheless, the data in Figure 14a show that azimuthal symmetry in the shock launched along the sidewalls of the cylinder is very good. A linear fit to position versus time data computed for each CFBG yields: 6.43 ± 0.030 mm/μs for CFBG1, 6.54 ± 0.022 mm/μs for CFBG2, 6.43 ± 0.033 mm/μs for CFBG3 and 6.42 ± 0.027 mm/μs for CFBG4. It is not clear whether these differences are due to azimuthal asymmetry of the initial explosive drive and ensuing hydrodynamics or if this is due sensor error. Cross comparison with complementary diagnostics and multi-dimensional hydrodynamic simulations would be necessary to resolve the cause of these differences. Taking all four CFBG shock position line plots and averaging, we show in Figure 14b the average shock position versus time. A linear fit to the CFBG data in Figure 14b is also shown, and the computed slope yields an average shock velocity of 6.46 ± 0.024 mm/μs. This value is slightly above the longitudinal speed of sound in aluminum 6061 ($c_{\mathrm{Al}}$ = 6.3 mm/μs) and is consistent with a weak shock propagating at a speed slightly above $c_{\mathrm{Al}}$.

## 4. Discussion

The results shown in the previous section demonstrate several examples of how CFBGs are utilized to measure detonation front and shock wave propagation dynamics. The choice between interrogation approaches, i.e., spectrally integrated versus time-streaked spectrally resolved, is dependent on the details of the expected loading applied to the CFBG. Our practice has been to use the economical spectrally-integrated technique when CFBG loading is expected to consume the entire CFBG length. The choice is based on a priori knowledge of the dynamics and processes that are expected in strong shock or detonation events that cause destructive processing of the CFBG with no chance of portions of the grating to remain reflective or intact after the passage of the shock or the detonation front. Under this type of loading, the spectrally-integrated approach yields accurate and unambiguous results, such as presented in the example of the detonation front measurements in PBX-9501 presented in Section 3.1. If, however, there is an expected drop off in shock pressure such that portions of the CFBG are expected to survive the shock with the possibility of elastic response in the CFBG either in tension or compression, then the time-streaked spectrally-resolved approach is preferred. However, when using the time-streak approach, there is a slight compromise in interrogation speed at 100 MHz compared with the ∼1-GHz bandwidth detection in the spectrally-integrated approach. Yet, despite this trade-off in interrogation speed between the two approaches, a nominal shock wave (or detonation front) traveling at 10 mm/μs is still sufficiently temporally resolved at slower 100-MHz interrogation since the shock transit in 10 ns is 100 μm, and typical tracking distances determined by the CFBG sensor can range from 10 mm–200 mm (see Table 1).

A second factor for consideration is spatial resolution. In previous papers [1,4], we discussed that in the spectrally-resolved approach, resolution is determined by a combination of the CFBG sensor properties (i.e., length, chirp and spectral band coverage), detector noise level and the recording oscilloscope digitizer vertical bit resolution. The recording bit resolution contributes to resolution because voltage resolution is tied to the smallest spectral change detectable induced by a change in length of the CFBG. From [4] an estimate of the spatial resolution for a 1-GHz “8-bit” (six ENOB—effective number of bits) at a digitizer sampled at 2.5 GS/s was 46 μm and 460 μm for a 10 mm and 100 mm-long C-band CFBG, respectively. The chirp rates of the CFBG used in [4] were 0.35 mm/nm and 3.5 mm/nm for the 10-mm and 100-mm CFBGs, respectively. Improvement in spatial resolution is achieved by changeover to a “12-bit” digitizer (Keysight M9703A AXIe digitizer) with 8.8 ENOB at a 1-GHz analog bandwidth. The data in Figure 6a from the results in Section 3.1 of this paper were recorded at 8.8 effective bits with a sample rate of 3.2 GS/s, and oversampling allowed 10 point averaging to lower our noise floor to ∼2.5 mV while still maintaining a temporal resolution of a few ns. This yields a spatial resolution of approximately 300 μm for the 120 mm-long CFBGs (chirp: 3.42 mm/nm) fielded on the PBX-9501 detonation front tracking test with the spectrally-integrated interrogation system. Slight improvement in resolution was achieved because of the increase in the ENOB from 6–8.8 in the switch over to a digitizer with better vertical resolution. Further improvement (2×) in the spatial resolution can be achieved by changing the chirp rate to 1.75 mm/nm. Such a CFBG would have a nearly 60-nm spectrum that would cover both C- and L-bands. We are currently investigating the availability of a phase mask to manufacture wide-band CFBGs of this type for an additional increase in resolution.

Spatial resolution in the time-streak spectrally-resolved approach was introduced in Section 2.1. The spatial resolution for a given CFBG is determined by the effective number of sample points in a single pulse time window (see Equation (2)). Although the bandwidth and chirp rate properties of the CFBG do not appear explicitly in Equation (2), the maximum total dispersion length, L${}_{D}$, is fixed for a given CFBG since the bandwidth and chirp rate limit the total fiber dispersion required to maintain the stretched pulse within the single pulse time window, Δt${}_{w}$ = 10 ns. Therefore, the CFBG bandwidth limits the maximum L${}_{D}$, which then determines spectral resolution R${}_{\lambda }$ according to Equation (2). CFBG spatial resolution is then calculated by multiplying R${}_{\lambda }$ by the chirp rate. For example, the 10 mm-long CFBG example displayed in Figure 3 with a calculated spectral resolution R${}_{\lambda }$ = 0.18 nm and 0.35 mm/nm chirp rate yields a spatial resolution of 63 μm. This value is quite comparable to the resolution of 46 μm for 10-mm CFBG in the spectrally-integrated approach. For the 120 mm-long CFBGs (chirp: 3.42 mm/nm) fielded on the PBX-9501 detonation front tracking test (Section 3.1), the spatial resolution was 620 μm, compared to 300 μm for the same CFBG type using 12-bit digitizer recording in the spectrally-integrated approach. Adjustment of the spatial resolution is possible by varying the total fiber dispersion length, CFBG chirp rate or by changing the effective bandwidth/speed of the recording system (which is currently limited to 25 GHz by the digitizing oscilloscope). Alternatively, if one is willing to trade-off the interrogation speed (time resolution) for improved spatial resolution, one may decrease the laser repetition rate to 50 MHz to allow for a longer 20-ns time slice window in which the total fiber dispersion can be increased to accommodate twice the effective number of points in a single pulse spectrum.

Finally, consideration of the FBG interrogator system cost is important. If experimental conditions are such that prompt destructive mode shock processing of the CFBG sensor is expected, the most economical solution is the spectrally-integrated approach. The ASE light source cost, detectors and recording speed requirements are modest with components readily available. In the time-streaked spectrally-resolved approach, the cost of the system is dominated by the high-speed four-channel recording oscilloscope, which can approach $150 K–200 K U.S. dollars. Further, GHz photoreceivers and ultrafast laser sources are also more costly, such that the cost per sensor channel is ∼4× greater in the time-streak approach. Our practice has been to deploy time-streak interrogator systems when warranted for FBG sensing when the speed and spectrally-resolved measurements are needed.

## 5. Conclusions

High speed CFBG-based sensing methodologies have been described for measuring detonation front and shock wave dynamics along the length of a single-mode CFBG. The lengths of CFBG sensors used for our shock physics applications range between 10 mm and 200 mm and principally operate in reflection in the C, L, or C + L band. The temporal and spatial resolution of these sensors depends on the properties of the CFBG and the interrogator instrumentation. For the shortest 10 mm-long CFBGs that have a chirp rate of 0.35 mm/nm, spatial resolution is approximately 50 μm, and for the longer CFBGs with a chirp rate of 3.5 mm/nm, spatial resolution is approximately several hundred microns. Two interrogation schemes were described. The first approach measures the time change of the spectrally-integrated light return after illumination by an ASE light source and offers a time resolution approaching a few ns. The second approach measures the full spectral response of the CFBG by using a 100-MHz 90-fs mode-locked coherent laser pulse to interrogate the CFBG. Pulses are then chromatically dispersed and time stretched to temporally streak the return spectrum of the CFBG. Time resolution in the time-streak spectrally-resolved approach is 10 ns. Both interrogation approaches are still sufficiently fast enough to resolve detonation and shock dynamics. Three experimental tests were described to demonstrate the operational characteristic of CFBG-based detonation front and shock wave sensors, and in some cases where the shock pressures are weak, elastic response of the CFBG was observed. If elastic response in the CFBG due to weak pressures is encountered, the time-streak spectrally-resolved approach is deemed as the preferred approach. We are continuing to further develop these fiber-based shock sensor systems by improving performance with a goal to achieve a spatial resolution below 10 μm.

## Figures and Tables

**Table 1:** Listing of the various CFBGs used in this work.

Length (mm)	Band	Chirp (mm/nm)	Coating
10	C	0.35	acrylate
100	C, C + L	3.5, 1.75	polyimide, acrylate
120	C	3.4	polyimide, acrylate
200	C, C + L	3.45	polyimide

**Table 2:** Listing of the diagnostic type and count fielded on the PBX-9501 slab detonation test.

Diagnostic	Sensor Count	CFBG Chirp (mm/nm) or Pin Spacing (mm)
CFBG	2 (120 mm)	3.41, 3.42
Time-Streak CFBG	2 (120 mm)	3.43, 3.42
Shorting Switch	16	10.5
Shorting Pin	8	25.4
Piezo Pin	8	25.4

**Table 3:** Detonation velocity results from the PBX-9501 slab test.

Diagnostic	Measured Velocity (mm/μs)
CFBG${}_{AVG}$	8.84 ± 0.014
Shorting Switch	8.78 ± 0.11
Shorting Pin	8.80 ± 0.04
Piezo Pin	8.80 ± 0.03

## Abbreviations

The following abbreviations are used in this manuscript:

FBG	Fiber Bragg grating
CFBG	Chirped fiber Bragg grating
PBX	Plastic bonded explosive
PDV	Photonic Doppler velocimetry
VISAR	Velocity interferometer for a system of any reflector
ASE	Amplified spontaneous emission
LUT	Look-up table
OBR	Optical backscatter reflectometer
LWG	Line wave generator
HE	High explosive
LoFi	Low fidelity
PMMA	Polymethylmethacrylate
ENOB	Effective number of bits
